# A Rare Case of Rapidly Progressing Merkel Cell Carcinoma

**DOI:** 10.7759/cureus.88816

**Published:** 2025-07-26

**Authors:** David Sau Ting Jain, Omer Riyadh, Kayla Tran, Nguyen L Ngo, Dei-Wook Kang

**Affiliations:** 1 Medicine, O'Connor Hospital, San Jose, USA; 2 Medicine, Kansas City University of Medicine and Biosciences, Joplin, USA; 3 Internal Medicine, O'Connor Hospital, San Jose, USA

**Keywords:** acute decompensated respiratory failure, cancer metastasis, distal metastasis, merkel cell carcinoma (mcc), oncology departement, tumor mass effect

## Abstract

Merkel cell carcinoma (MCC) is a highly aggressive neuroendocrine tumor, known for its rapid progression and poor prognosis. It most commonly affects elderly and immunocompromised individuals, with variable clinical presentations that can make diagnosis challenging. MCC is often mistaken for other skin malignancies, such as basal or squamous cell carcinoma, or even benign skin lesions. Early diagnosis is critical to initiate appropriate treatment, typically involving surgery, radiation, and immunotherapy. This report describes a 66-year-old man diagnosed with MCC with regional lymph node involvement. Immunotherapy was started promptly; however, within a year, the disease progressed to the mediastinum, involving the esophagus and trachea, an uncommon site of metastasis. The patient ultimately died in the ICU from respiratory failure. This case highlights the aggressive behavior of MCC and underscores the importance of vigilant follow-up, even after early diagnosis. Once metastasis to the lymph nodes occurs, MCC becomes particularly challenging to manage, especially when it follows an atypical metastatic pattern.

## Introduction

Merkel cell carcinoma (MCC) is a rare but highly aggressive neuroendocrine malignancy of the skin, originally described for its nest-like or “trabecular” pattern and neurosecretory granules. Although it constitutes fewer than 1% of all cutaneous tumors, the incidence of MCC has risen globally, partly due to improved diagnostic awareness and an aging population with significant ultraviolet (UV) exposure or compromised immune systems [1,2]. Clinically, MCC often appears on sun-exposed areas, such as the face, scalp, and neck, as a rapidly enlarging, firm, painless nodule that may be mistaken for other skin lesions, including basal or squamous cell carcinoma and melanoma [1,3].

The pathogenesis of MCC commonly follows one of two main routes: (1) virus-mediated tumorigenesis via the integration of Merkel cell polyomavirus (MCPyV) and its T antigens or (2) high mutational burden resulting from UV-induced DNA damage in tumor suppressor genes like RB1 and TP53 [4,5]. Despite these divergent pathways, both MCPyV-associated and MCPyV-negative MCC share a propensity for regional lymph node involvement and distant spread [6]. Notable risk factors include immunosuppression, advanced age, extensive sun exposure, and comorbid conditions, all of which can accelerate disease progression [1,7].

Diagnosis typically requires histopathological confirmation, supported by immunohistochemical staining for cytokeratin 20, chromogranin, and synaptophysin [1,8]. Clinicians often perform sentinel lymph node biopsy (SLNB) and imaging studies for accurate staging, and wide local excision with appropriate margins is recommended for localized lesions [3,9]. Adjuvant radiotherapy can help reduce locoregional recurrence, particularly in high-risk scenarios. For advanced or metastatic cases, immunotherapies targeting PD-1/PD-L1 (such as pembrolizumab and avelumab) have demonstrated robust response rates and durable disease control, reshaping the therapeutic landscape of MCC [2,10].

In this case report, we describe a 66-year-old male patient with multiple comorbidities, including diabetes mellitus, chronic kidney disease, and ischemic heart disease, who initially presented with a rapidly enlarging lesion on the right thigh and subsequently developed distal metastasis to the mediastinum. Histopathological analysis and immunostaining confirmed MCC, underscoring the importance of high clinical suspicion for uncommon cutaneous tumors, especially in older or immunocompromised populations. Early recognition and prompt, multidisciplinary management are crucial for improving outcomes in this aggressive skin cancer.

## Case presentation

We present the case of a 66-year-old man with a past medical history significant for type 2 diabetes, hyperlipidemia, and hypertension, who was diagnosed with MCC in July 2023, with metastasis to the regional lymph nodes. The primary lesion was initially identified on the right thigh, accompanied by extensive lymphadenopathy involving the right iliac chain, groin, and retroperitoneal regions. The patient was subsequently started on outpatient immunotherapy, receiving one cycle of pembrolizumab, two cycles of avelumab, and three cycles of carboplatin with etoposide. However, he did not tolerate chemotherapy well, experiencing significant side effects, including severe neutropenia, which necessitated multiple treatment interruptions.

**Figure 1:**
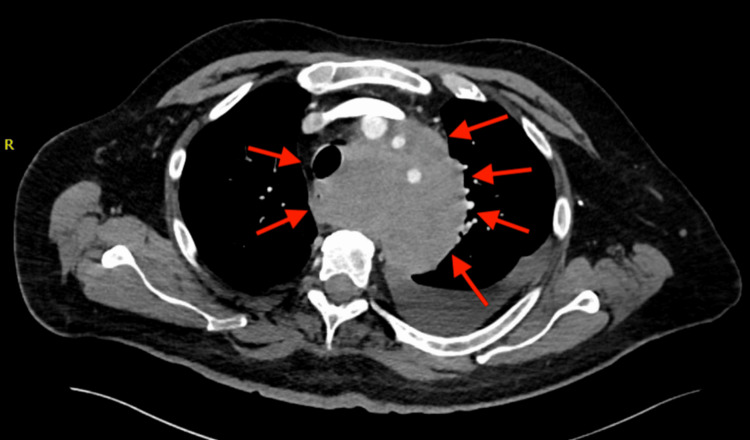
CT scan performed on July 31, 2024, revealing a newly discovered mediastinal mass (red arrows)

A CT scan performed on July 31, 2024, demonstrated further disease progression, revealing a newly developed large soft tissue lesion measuring 10.4 × 9.0 × 9.0 cm with irregular borders involving the perivascular space, an abnormality not present on imaging at the time of the initial MCC diagnosis (Figure 1). The mass encased the aortic arch, left common carotid artery, and left subclavian artery. Additionally, the esophagus and trachea were displaced to the right of the midline. Due to disease progression, the oncologist determined that the patient was no longer a candidate for further chemotherapy and recommended consideration of palliative care.

On August 14, 2024, the patient presented to the emergency department (ED) after falling in the bathroom while showering. He reported slumping to the ground due to generalized weakness while standing in the shower.

A complete blood count (CBC) upon admission showed low hemoglobin and hematocrit, likely secondary to immunotherapy treatment. A comprehensive metabolic panel (CMP) revealed elevated blood urea nitrogen (BUN) and creatinine levels. A fecal occult blood test (FOBT) was positive, although there were no overt signs of gastrointestinal bleeding. The patient's hemoglobin levels stabilized after receiving two packed red blood cell (PRBC) transfusions.

On physical examination, the patient showed moderate use of accessory respiratory muscles and spoke with a muffled voice, needing to pause for breath after each sentence. His oxygen saturation ranged from 91% to 94% while receiving 2-4 L of oxygen via nasal cannula. Additionally, he exhibited generalized weakness and bilateral lower extremity edema.

**Figure 2:**
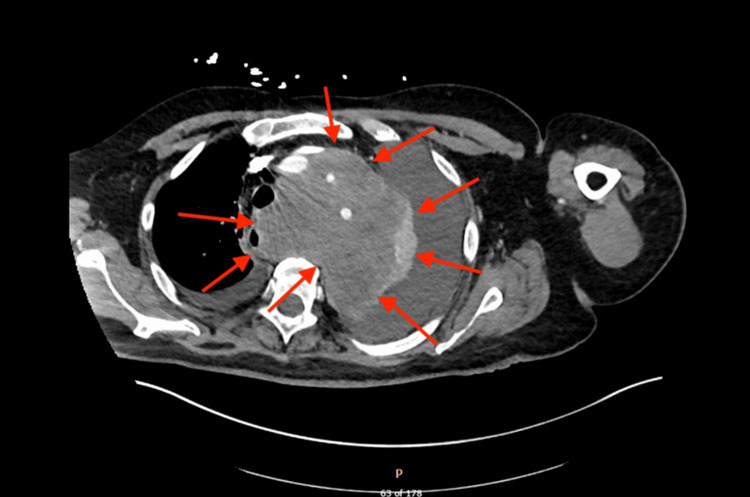
CT scan performed on August 31, 2024, revealing a mediastinal mass that has encased structures in the surrounding tissues

**Figure 3:**
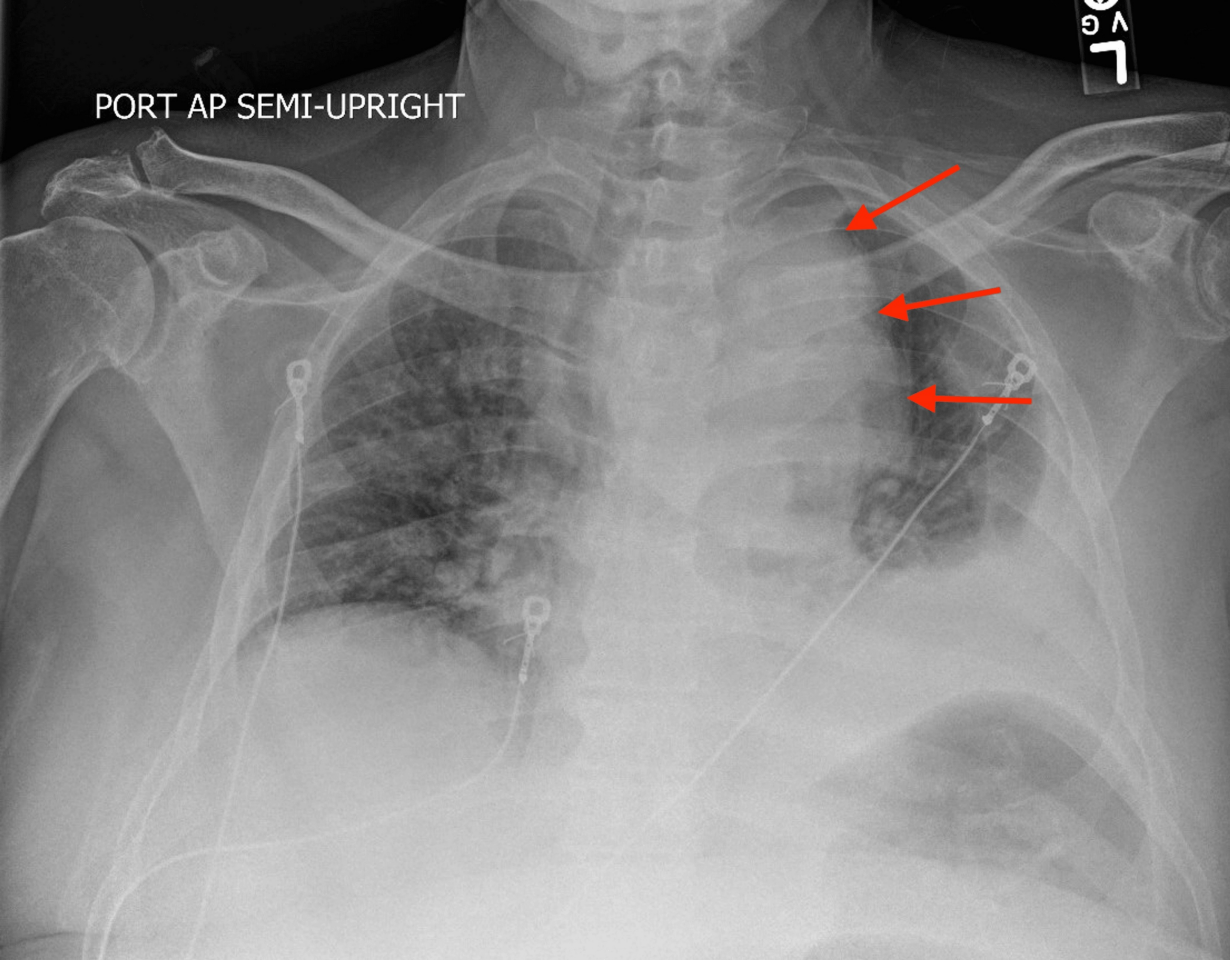
Mediastinal mass encasing the aortic arch, as seen on initial ED presentation on August 13, 2024

A CT scan performed on August 23, 2024, showed a moderate left pleural effusion with left basilar atelectasis and a small right pleural effusion. The dense soft tissue mass in the superior mediastinum had increased in size, now measuring 12.6 × 11.4 × 10.1 cm. Both the CT scan (Figure 2) and chest X-ray (Figure 3) demonstrated extensive mediastinal involvement and upper respiratory invasion by metastatic cancer. Palliative care was discussed with the patient and family, given the poor disease prognosis; however, the patient declined and expressed a desire to continue aggressive treatment.

The patient’s respiratory function continued to deteriorate, leading to his admission to the intensive care unit (ICU) for respiratory failure, where he required BiPAP therapy. Palliative care was revisited with the patient and family. He ultimately died on August 26, 2024, due to respiratory failure.

## Discussion

MCC is a rare neuroendocrine tumor, with an average of only 2,500 cases per year in the United States [11]. It is typically associated with UV radiation, immunosuppression, and MCPyV. Early neoplasms often lack distinct characteristics and can easily be mistaken for other skin lesions [12]. The head and neck are the most common regions for primary MCC, accounting for 48%-53% of cases [13].

Unfortunately, due to its rapid progression, 31% of patients present with lymphovascular involvement at the time of diagnosis, and the rate of early metastasis or distant organ involvement reaches 100% within five years [14]. MCC is considered the most lethal skin cancer after melanoma [15]. The majority of distant metastases occur in the abdomen, particularly involving the abdominal viscera, liver, and pancreas [16]. Extra-abdominal metastases are relatively uncommon, most frequently affecting the vertebral column and lungs [14].

This case is notable for distant metastasis to the upper mediastinum, despite the primary lesion being located in the lower extremity. While thoracic lymph node involvement is not uncommon, it is typically associated with primary lesions arising in the head and neck region. In this patient, the extensive mediastinal and pulmonary metastases represented an atypical pattern of spread, ultimately leading to acute respiratory failure and death.

Due to the rarity of this disease, establishing optimal treatment strategies remains a challenge. Wide surgical excision is recommended for primary lesions, though no specific margin has been definitively associated with a decreased risk of recurrence [14]. Additionally, few studies address the treatment of recurrent lesions. However, radiotherapy has shown promising results, with a five-year local relapse-free rate of 90% [17]. This approach is particularly beneficial in cases with regional lymph node metastasis or when surgical excision would require extensive reconstruction [17]. Patients undergoing radiotherapy may experience cutaneous desquamation and fatigue [14]. However, if the disease has already progressed to involve distant lymph node metastasis, immunotherapy is recommended as the first-line treatment, in accordance with the NCCN Clinical Practice Guidelines in Oncology [18].

These considerations are increasingly important, as MCC progresses rapidly in the elderly and immunocompromised populations. Elderly patients, in particular, have reduced tolerance for multimodal treatment, making the selection of an appropriate treatment plan critical for improving outcomes and maintaining quality of life [14].

## Conclusions

This case illustrates the aggressive nature of MCC and the significant challenges in managing this highly lethal neuroendocrine tumor, which is associated with poor prognosis and high mortality. Despite advances in therapeutic protocols, the patient ultimately succumbed to the disease, which was complicated by an atypical metastatic pattern and rapid progression.

Early recognition and treatment of MCC are crucial, especially in elderly and immunocompromised patients. This case underscores the need for continued research to improve diagnostic accuracy and optimize therapeutic strategies for this rare but aggressive malignancy.
